# Cutaneous squamous cell carcinoma in patients with solid organ malignancy

**DOI:** 10.1016/j.jdin.2023.12.003

**Published:** 2023-12-29

**Authors:** Chandler Johnson, Morgan Groover, Emily Granger, Fadi Murad, Emily Karn, Emily S. Ruiz

**Affiliations:** a Medical College of Georgia at Augusta University, AU/UGA Medical Partnership, Athens, Georgia; b Department of Dermatology, Brigham and Women’s Hospital, Boston, Massachusetts

**Keywords:** clinical research, cutaneous squamous cell carcinoma, oncology, solid organ malignancy

*To the Editor:* Immunosuppression is associated with impaired tumoricidal activity that confers an increased risk of cutaneous squamous cell carcinoma (cSCC).^1^^,^^2^ The association of immunosuppression due to organ and stem cell transplant, and hematopoietic malignant disease and cSCC outcomes is well defined.^3^ Patients with solid organ malignancy (SOM) are considered to have some degree of immunosuppression within the tumor microenvironment, though this is a complex ecosystem with constantly evolving clinical and prognostic significance.^4^ However, the relationship between SOM and cSCC risk has not been documented. The objective of this study is to evaluate the incidence, tumor characteristics, and outcomes of cSCC in patients with SOM compared to patients with no history of SOM.

Patients with SOM (*n* = 745) and patients with no history of solid organ or hematologic malignancy (*n* = 2748) were identified from an institutional review board–approved cohort of cSCCs treated at Brigham and Women’s Hospital (BWH) between 2010 and 2017. Patient demographics, tumor characteristics, treatments, and outcomes are outlined in Table I. The total number of tumors in the SOM and no history of solid organ or hematologic malignancy group were 807 and 4496, respectively. Patients with SOM had a greater incidence of high-stage tumors (BWH stage T2b and above) as defined by the BWH tumor classification system for cSCC (SOM: 51 (6.3%), no history of solid organ or hematologic malignancy: 67 (1.49%) *P* ≤ .001). There were no differences in the frequency of local recurrence, in transit metastasis, nodal metastasis, distant metastasis, or disease-specific death (Table I). We conducted a multivariate analysis to examine the association between SOM and poor outcomes. Based on univariate analysis, models were adjusted for age, gender, and BWH stage. However, the findings did not reveal significant associations between history of SOM and development of poor outcomes (Supplementary Table I, available via Mendeley at https://doi.org/10.17632/v4cvwt8pzr.1).

**Table I:** Patient (solid organ malignancy *n* = 745, no history of solid organ or hematologic malignancy *n* = 2748) and tumor (solid organ malignancy *n* = 807, no history of solid organ or hematologic malignancy *n* = 4496) characteristics by history of solid organ malignancy

Patient and tumor characteristics	Solid organ malignancy (*n* = 745)	No history of solid organ malignancy (*n* = 2748)	*P* value
Age at diagnosis of cSCC, median y (IQR)	73.52 (67.04-80.05)	71.29 (62.95-80.13)	<.001
Male sex, *n* (% of patients)	405 (54.4)	1414 (54.3)	.16
Type of solid organ malignancy, *n* (% of patients)∗	1158	-	*n/a*
Breast	230 (19.8)	-	
Prostate	253 (21.8)	-	
Lung	103 (8.9)	-	
Head and neck	51 (4.4)	-	
Bladder	262 (22.62)	-	
Kidney	45 (3.88)	-	
Colorectal	95 (8.2)	-	
Other	119 (10.27)	-	
Immunosuppressed, *n* (% of patients)	127 (17.05)	288 (10.48)	<.001
Tumor diameter, *n* (% of tumors)			.49
Less than 2 cm	709 (87.86)	3987 (88.68)	
Greater than 2 cm	98 (12.14)	509 (11.32)	
Depth of invasion, *n* (% of tumors)			.26
Dermis/subcutaneous fat	777 (96.28)	4362 (97.02)	
Beyond subcutaneous fat	30 (3.72)	134 (2.98)	
Differentiation, *n* (% of tumors)			.09
Well	707 (87.61)	3850 (97.02)	
Moderate	43 (5.33)	295 (6.72)	
Poor	57 (7.06)	244 (5.56)	
LCNI, *n* (% of tumors)	16 (1.98)	55 (1.22)	.08
BWH stage, *n* (% of tumors)†			<.001
T1	673 (83.4)	3568 (79.36)	
T2a	83 (10.29)	861 (19.15)	
T2b	46 (5.7)	42 (0.93)	
T3	5 (0.62)	25 (0.56)	
Number of patients with range of number of cSCCs, *n* (% of patients)			.29
1-5 cSCCs	711 (95.4)	2655 (96.7)	
6-10 cSCCs	25 (3.4)	66 (2.4)	
>10 cSCCs	9 (1.2)	27 (0.98)	
Treatment, *n* (% of tumors)			.58
Excision	288 (35.69)	1565 (34.81)	
Mohs micrographic surgery	421 (52.17)	2425 (53.96)	
Other	98 (12.14)	505 (11.23)	
Outcomes, *n* (% of tumors)			
Local recurrence	11 (1.36)	53 (1.18)	.65
In transit metastasis	5 (0.62)	16 (0.36)	.27
Nodal metastasis	10 (1.24)	42 (0.93)	.41
Distant metastasis	4 (0.5)	14 (0.5)	.41
Death due to disease	5 (0.62)	24 (0.53)	.76

*BWH*, Brigham and Women’s Hospital; *cSCC*, cutaneous squamous cell carcinoma; *LCNI*, large caliber nerve invasion.

∗ Includes patients who had greater than 1 solid organ malignancy.

† Tumor staging was determined using Brigham and Women’s cSCC staging criteria.

At the time of cSCC diagnosis, 89 (11.9%) patients with SOM were undergoing active oncologic treatment including chemotherapy, immunotherapy, radiation, or a combination of these modalities. There were no distinctions in tumor characteristics or outcomes observed between those receiving active oncologic treatment for SOM and those who were not (Table II). For patients with SOM, active treatment for their malignancy did not seem to be related to a greater incidence of high-stage tumors or disease outcomes.

**Table II:** Tumor characteristics and outcomes by active treatment for solid organ malignancy∗

Tumor characteristics	Active treatment (*n* = 89)	Not active treatment (*n* = 718)	*P* values
Diameter, millimeters			.45
Less than 2 cm	76 (85.39)	633 (88.16)	
Greater than 2 cm	13 (14.61)	85 (11.84)	
Depth of invasion, *n* (%)			.62
Dermis/subcutaneous fat	87 (97.75)	695 (96.8)	
Beyond subcutaneous fat	2 (2.25)	23 (3.2)	
LCNI, *n* (%)	5 (83.33)	11 (35.48)	.03
Differentiation, *n* (%)			.26
Well	75 (84.27)	632 (88.02)	
Moderate	8 (8.99)	51 (7.1)	
Poor	6 (6.74)	35 (4.87)	
Tumor staging			.61
T1	74 (83.15)	599 (83.43)	
T2a	7 (7.87)	76 (10.58)	
T2b	7 (7.87)	39 (5.43)	
T3	1 (1.12)	4 (0.56)	
Active therapy for solid organ malignancy and squamous cell carcinoma outcomes			
Active therapy type, *n* (%)		-	
Systemic therapy†	80 (89.8)	-	
Radiation	5 (5.6)	-	
Systemic therapy & radiation	4 (4.4)	-	
Outcomes, *n* (%)			
Local recurrence	0 (0.0)	11 (1.53)	.24
In transit metastasis	1 (1.12)	4 (0.56)	.52
Nodal metastasis	2 (2.25)	8 (1.11)	.36
Distant metastasis	1 (1.12)	3 (0.42)	.37

*LCNI*, Large caliber nerve invasion.

∗ Tumor staging was determined using Brigham and Women’s cutaneous squamous cell carcinoma staging criteria.^5^

† Systemic therapy refers to treatment with chemotherapy or immunotherapy.

The findings of this study indicate an elevated incidence of high-stage cSCC among individuals with a prior history of SOM. However, no definitive associations were established been SOM and adverse outcomes. Additional research is warranted to thoroughly examine the interplay between SOM, cSCC tumorigenesis, and disease outcomes.

## Conflicts of interest

Dr Ruiz has served as a consultant for Regeneron, Genetech, and Checkpoint Therapeutics. Author Johnson, Author Groover, Author Granger, Dr Murad, and Author Karn have no conflicts of interest to declare.

## References

[1] Ishitsuka Y., Hanaoka Y., Tanemura A., Fujimoto M. (2021). Cutaneous squamous cell carcinoma in the age of immunotherapy. Cancers.

[2] Kim C., Cheng J., Colegio O.R. (2016). Cutaneous squamous cell carcinomas in solid organ transplant recipients: emerging strategies for surveillance, staging, and treatment. Semin Oncol.

[3] Tam S., Yao C.M.K.L., Amit M. (2020). Association of immunosuppression with outcomes of patients with cutaneous squamous cell carcinoma of the head and neck. JAMA Otolaryngol Head Neck Surg.

[4] Giraldo N.A., Sanchez-Salas R., Peske J.D. (2019). The clinical role of the TME in solid cancer. Br J Cancer.

[5] Jambusaria-Pahlajani A., Kanetsky P.A., Karia P.S. (2013). Evaluation of AJCC tumor staging for cutaneous squamous cell carcinoma and a proposed alternative tumor staging system. JAMA Dermatol.

